# Integration of Dental Implants in Conjunction with EDTA-Conditioned Dentin Grafts: An Experimental Study

**DOI:** 10.3390/dj9060063

**Published:** 2021-06-01

**Authors:** Payam Farzad, Ted Lundgren, Adel Al-Asfour, Lars Andersson, Christer Dahlin

**Affiliations:** 1 Department of Biomaterials, Institute for Surgical Sciences, The Sahlgrenska Academy, University of Gothenburg, 405 30 Gothenburg, Sweden; payamfarzad@hotmail.com; 2 Department of Oral & Maxillofacial Surgery, Karolinska University Hospital Stockholm, 405 30 Gothenburg, Sweden; 3 Department of Pediatric Dentistry, Institute of Odontology, The Sahlgrenska Academy, University of Gothenburg, 405 30 Gothenburg, Sweden; ted.lundgren@odontologi.gu.se; 4 Department of Surgical Sciences, Faculty of Dentistry, Kuwait University, Safat 12037, Kuwait; adelalsfour@hsc.edu.kw; 5 Department of Oral & Maxillofacial Surgery, Faculty of Odontology, Malmö University, 205 06 Malmö, Sweden; dr.lars.andersson@gmail.com; 6 Department of ENT and Oral & Maxillofacial Surgery, NU Hospital Organization, 461 30 Trollhättan, Sweden

**Keywords:** grafted dentin, dental implants, EDTA, experimental study

## Abstract

This study was undertaken to investigate the integration of titanium micro-implants installed in conjunction with previously dentin-grafted areas and to study the morphological appearance, mineral content, and healing pattern of xenogenic EDTA-conditioned dentin blocks and granules grafted to cavities in the tibial bone of rabbits. Demineralized and non-demineralized dentin blocks and granules from human premolars were implanted into cavities prepared on the lateral aspects of the tibias of rabbits. After a healing period of six months, micro-implants were installed at each surgical site. Histological examinations were carried out after 24 weeks. Characterization of the EDTA-conditioned dentin blocks was performed by means of light microscopy, dental X-rays, scanning electron microscopy, and energy dispersive X-ray analysis (EDX). No implants were found to be integrated in direct contact with the dentin particles or blocks. On the EDTA-conditioned dentin surface, the organic marker elements C and N dominated, as revealed by EDX. The hydroxyapatite constituents Ca and P were almost absent on the dentin surface. No statistically significant difference was observed between the EDTA-conditioned and non-demineralized dentin, as revealed by BIC and BA. The bone-inductive capacity of the dentin material seemed limited, although demineralization by means of EDTA indicated higher BIC and BA values in conjunction with the installed implants in the area. A 12 h EDTA treatment did not fully decalcify the grafts, as revealed by X-ray analysis.

## 1. Introduction

With improved titanium surface properties, rehabilitation with dental implants has improved considerably over time. The time to obtain osseointegration is shortened, and secondary stability is improved [1]. Use of dental implants is frequently restricted due to the physiological atrophy of the alveolar process following tooth loss [2,3].

Bone augmentation of the severely resorbed site is a prerequisite for facilitating dental implant placement. However, autogenous bone is considered the gold standard among the various types of bone graft materials, although there are shortcomings such as donor site morbidity, limitations in the quantity of available bone, the prolongation of surgery, and treatment costs. For this reason, active research on new bone graft materials with a bone regeneration ability equivalent to autogenous bone but without the limitations of allogenic, xenogenic, and synthetic bone is constantly ongoing.

It is well known that, as a result of tooth avulsion and replantation as well as injury to the periodontal membrane, the root may be fused to the bone (ankylosis) and may be replaced by bone (osseous replacement).

In order to possibly modify the treatment protocols and to explore possible cost–benefit alternatives to commercially available bone replacement materials, there has been increased interest in exploring the use of human dentin as a source of graft material [4,5,6,7,8,9,10,11,12]. It has been shown that dentin possesses both osteo-inductive assets due to its content of certain non-collagenous proteins as well as osteoconductive properties [6,13,14]. This may indicate that dentin may function as a bone substitute in clinical settings. Previous studies have demonstrated that non-treated dentin grafted into non-osteogenic environments possesses only minimal bone-formation capacity [15,16,17].

When dentin is placed in direct contact with a native cortical bone, the novel bone formation seems to have more of an osteoconductive resorption characteristic [18]. Several studies propose preconditioning of the dentin block surface in order to facilitate release of BMP from the dentin [14,16,19,20,21]. Little is found in the literature regarding descriptive analysis of the preconditioned dentin blocks prior to graft transplantation. Secondly, knowledge of the tissue dynamics of dental implant integration when placed in areas grafted with dentin is still not fully understood.

Hence, the aims of this study were to investigate the morphological appearance and mineral content in preconditioned dentin grafts, to study the healing pattern of xenogenic demineralized dentin blocks and granules grafted onto cavities created in the tibial bone of rabbits, and to analyze the integration of titanium micro-implants installed in previously dentin-grafted areas.

## 2. Materials and Methods

The experimental model and set up used in the present study have previously been described by Farzad (2017) [22].

### 2.1. Animals and Anesthesia

Twelve 6-month-old New Zealand, male, white rabbits were used in the experiments. The experiments were carried out at the Animal Research Centre, Health Sciences Centre, Kuwait University. Thirty minutes prior to the experimental surgery, the rabbits were sedated with xylazine HCl (Rompun, Bayer, Leverkusen, Germany) at a dose of 5 mg/kg by intramuscular injection. The animals were anesthetized by intravenous injection of 35 mg/kg of ketamine HCl (Tekan, Hikma, Amman, Jordan). The protocol for animal experimentation was reviewed and approved by the Animal Research Centre of the Health Sciences Center, Kuwait (2015), and was strictly adhered to. A veterinarian was designated and responsible for administering the sedation and anesthesia and for the intra- and postoperative care of the animals. The animals were kept in separate cages and fed pellets and water ad libitum throughout the duration of the study.

### 2.2. Preparation of Dentin Grafts for Surgery

The dentin grafts were taken from human premolars, which had been extracted on orthodontic indications. The teeth (*n* = 24) were prepared in the following manner: The coronal part of the tooth was cut and removed with the help of rotary instruments. A total of 48 cylindrical dentin block grafts were prepared in standardized sizes by using a trephine bur, which was 5 mm in diameter, and the dentin block grafts were cut and trimmed to 2 mm thick dentin blocks by using a diamond dish. The thickness (2 mm) was checked with a caliper. The second half of the blocks (*n* = 24) were cut into granules to sizes of 1–3 mm. The dentin blocks and granules were placed in chlorhexidine for 60 min to reduce bacterial growth and were stored dry for one month. Twelve hours prior to grafting, the dentin grafts were rinsed in saline and half of the specimens were demineralized on their surfaces and placed in 24% EDTA, pH 7, for 12 h.

### 2.3. Preparation of Dentin Grafts for Histology, X-ray, and Elemental Analysis

Twelve dentin blocks were prepared as described (vide supra). Conventional dental X-rays (0.06 s, f 22.5 cm) were taken on all graft samples prior to decalcification. All samples were conditioned in 24% EDTA neutral, pH 7, for 12 h followed by a second X-ray analysis. Four samples were chosen for conventional SEM and energy dispersive X-ray analysis (EDX) in both the image mode and the element analysis mode. Four arbitrary dentin surfaces were analyzed, giving means and standard deviations of the elemental contents. Semiquantitative data for the elements C, N, O, P, Ca, and Au (100% in total) were sampled for 25 min.

### 2.4. Baseline Surgery—Graft Preparation and Placement

The surgical areas were shaved and washed with 7.5% iodine solution, and the animals were prepared for surgery. Local anesthesia composed of 1 mL lidocaine hydrochloride 2% + epinephrine 1:100,000 (Lignospan standard, Septodont, Saint Maur des Fosses Cedex, France) was administered in each experimental area. Bilateral incisions 2–3 cm long over the lateral aspect of the tibia were performed, and the tibial bone was exposed by surgical dissection. Two cavities 5 mm in diameter were prepared on the lateral tibia by using a trephine bur, penetrating through the tibial cortex. On the right tibia, the first cavity was filled with demineralized dentin block, and the second cavity was filled with demineralized dentin granules. On the left tibia, non-demineralized dentin was used in the same manner. No membrane or any other type of fixation was used. The soft tissue was sutured at two layers: the periosteum/muscle and the dermis. To compensate for perioperative and postoperative dehydration, 10 mL of sterile saline solution was injected subcutaneously immediately following surgery according to a previous protocol [23], and 50 mg/kg of antibiotics (Pen-Hista-strep, Vetoquinol SA, Lure Cedex, France) was administered by intramuscular injection. Antibiotic administration continued during the first 3 days after surgery. The rabbits were under frequent surveillance during the postoperative period.

### 2.5. Second Surgery—Implant Placement

**Figure 1:**
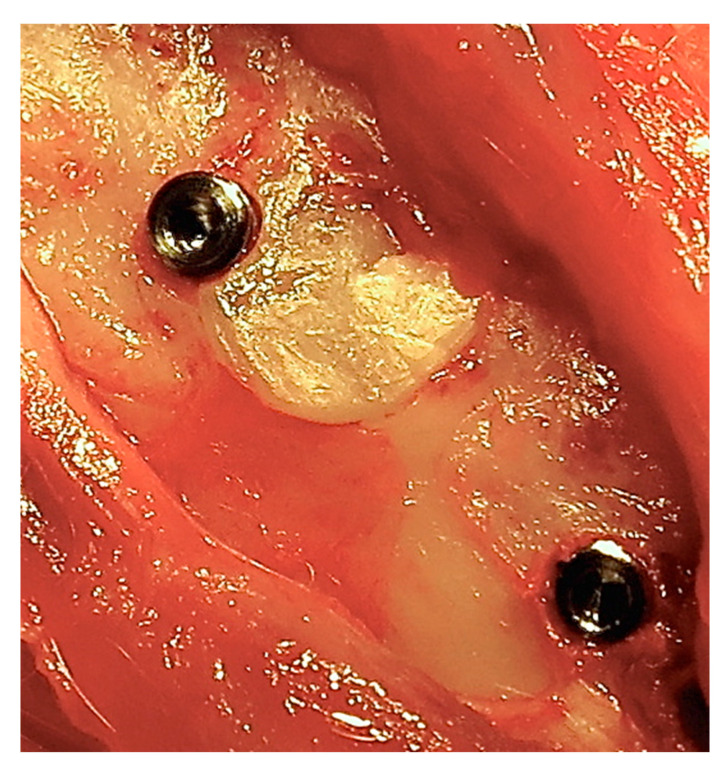
Micro-implants placed at the grafted sites. Top, dentin granulae; bottom, dentin block.

After a healing period of 24 weeks, the rabbits were anesthetized once again as described earlier. Surgical access was accomplished in a similar way, and one micro-implant (5 mm long and 2 mm in diameter), which was machined from medical-grade Ti (grade IV) rods (Elos, Pinol, Gørløse, Denmark), was installed at each surgical site in such a way that the coronal part of the micro-implant was placed in dentin (Figure 1). Soft tissue closure and postoperative care were identical to that during the baseline surgery. All rabbits were sacrificed 24 weeks after the second surgery by an overdose of ketamine, and block biopsies were prepared.

### 2.6. Histological and Histomorphometric Analysis

**Figure 2:**
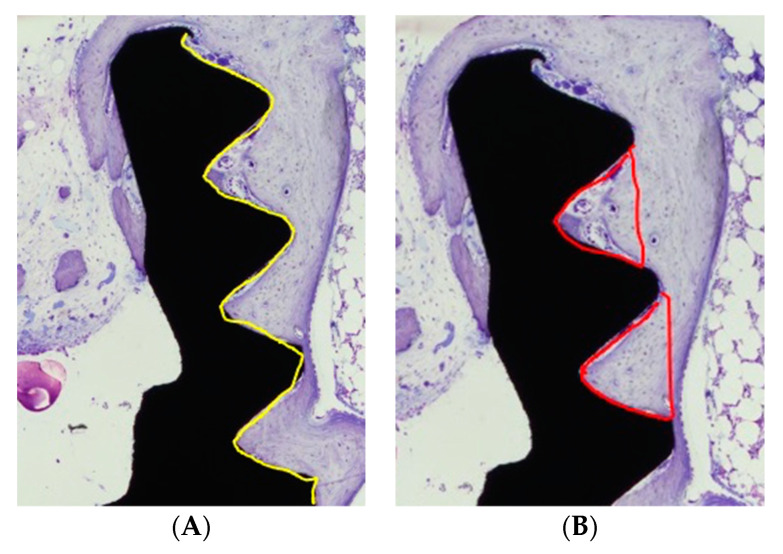
(**A**) Micro-implant subjected to histomorphometric analysis. The yellow line illustrates the region of interest for the BIC (bone-to-implant-contact) analysis (toluidine blue stain, ×10). (**B**) Micro-implant subjected to histomorphometric analysis. The marked red areas indicate the region of interest for BA (Bone Area) analysis (toluidine blue stain, ×10).

Following surgical removal en bloc, the samples were immersed and fixed in 10% neutral buffered formaldehyde, as described elsewhere (Alberius et al. 1989). We briefly describe it as follows: the fixated specimens were dehydrated in a graded series of ethanol, infiltrated with plastic resin, and polymerized prior to cutting along the long axis of the implant. A central ground section was prepared by cutting and grinding and was subsequently stained with toluidine blue. A region of interest (ROI) was defined and corresponded to the five coronal threads where dentin blocks or granules were placed. The specimens were observed along the five coronal threads. Samples of the non-mineralized dentin (*n* = 12) were compared with the EDTA-conditioned dentin samples (*n* = 14). The measurements of bone-to-implant contact (BIC) and the bone fill area (BA) within the threads were calculated on the mesial and distal aspects of each specimen. A mean value was then calculated for each specimen. The dentin, the bone-to-implant contact, and the relative amount of bone and dentin within the threads were determined using light microscopy (Nicon Eclipse E600) at 10 times magnification. The specimens were assessed using NIS Elements Microscope Imaging Software, Nikon. (Figure 2A,B).

### 2.7. Statistical Evaluation

Statistical analysis was performed using SPSS Ver 11.5 (SPSS Inc., Chicago, IL, USA). Wilcoxon signed rank test was used to compare the groups. All *p*-values of <0.05 were considered statistically significant.

## 3. Results

Four rabbits died during the healing period. The remaining eight rabbits recovered uneventfully and gained weight. Soft and hard tissue healing in all eight rabbits was uneventful, and there were no signs of infection. Hence, 32 micro-implants were available for analysis (demineralized group *n* = 16, non-demineralized *n* = 16).

### 3.1. Descriptive Characterization of Dentin Block

**Figure 3:**
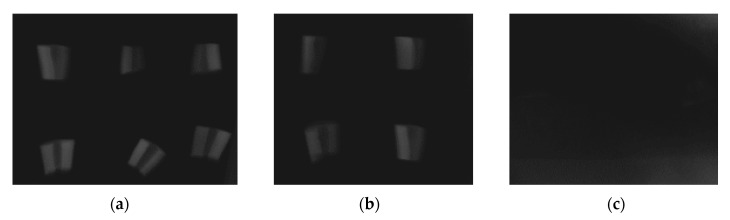
(**a**) Dental radiographic image of the dentin samples prior to EDTA conditioning. (**b**) Dentin after 12 h surface decalcification exhibits no visible change in image contrast. (**c**) After 4 weeks of decalcification, the dentin grafts are no longer discernible in the images.

**Figure 4:**
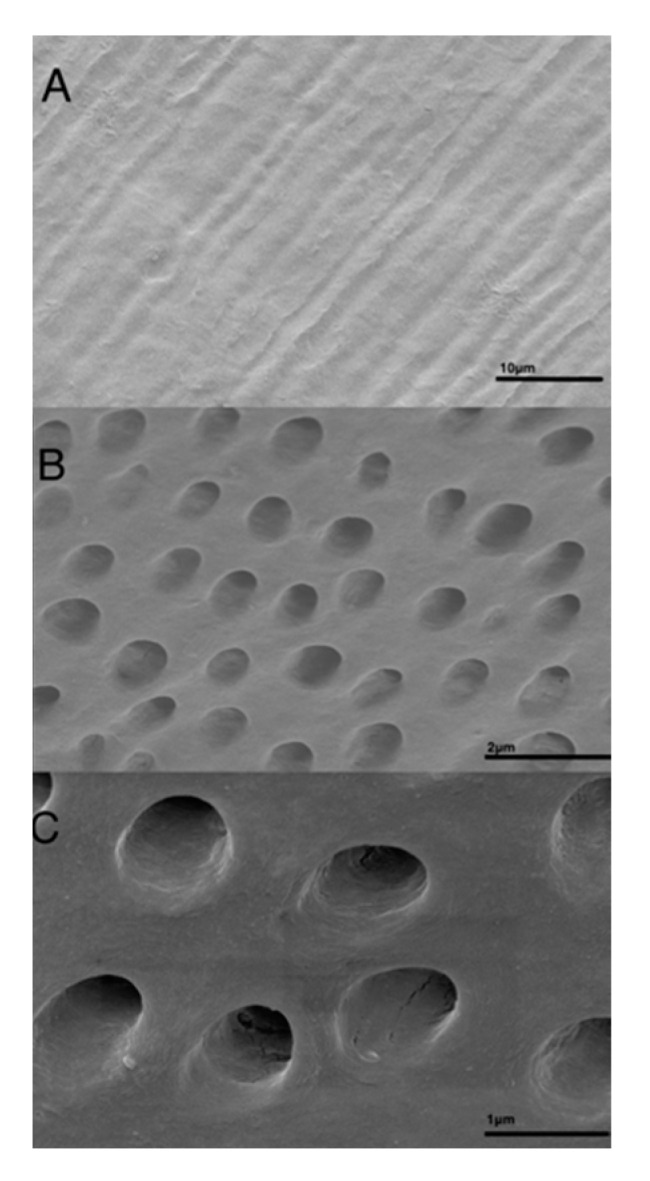
(**A**). An SEM image of the dentin graft exhibiting longitudinally sectioned tubules, ×2.7·10^3^. (**B**). Transversal section of dentin showing a tubule where the openings are devoid of cell processes, ×8.3·10^3^. (**C**). Close up of the tubules with regular peritubular walls and irregular inter-tubular dentin, ×19.8·10^3^.

In radiographic images taken prior to decalcification, the dentin grafts were clearly discernible (Figure 3a). After 12 h of EDTA surface conditioning, no differences in image contrast could be observed (Figure 3b). After 4 weeks of decalcification, the dentin grafts could not be discerned in the images (Figure 3c). In longitudinal sections of the dentin grafts, densely packed tubules, approximately 3 µm wide, were observed by means of SEM (Figure 4A). In transversal sections, tubules of approximately Ø 3 µm, devoid of odontoblast processes, were observed (Figure 4B). In transversal close ups, the dense peri-tubular walls were discerned, between which the demineralized collagenous inter-tubular dentin surface appeared (Figure 4C). On the demineralized dentin surface, the organic markers carbon (C) (55.13% ± 2.08) and nitrogen (N) (26.59% ± 2.00) dominated, as revealed by the EDX image analysis. The hydroxyapatite constituents calcium (Ca) (0.28% ± 0.14) and phosphorus (P) (0.00% ± 0.24) were close to being devoid on the dentin surface. Oxygen (O) (14.89% ± 0.38) remained present to some extent. Remnants of the gold (Au) surface coating (3.10% ± 0.75) used during SEM and EDX were also observed (Table 1).

On the calcified dentin surfaces, the organic markers carbon (C) and nitrogen (N) dominate. The inorganic elements calcium (Ca) and phosphorous (P) are close to being devoid after decalcification.

### 3.2. Bone Healing

**Figure 5:**
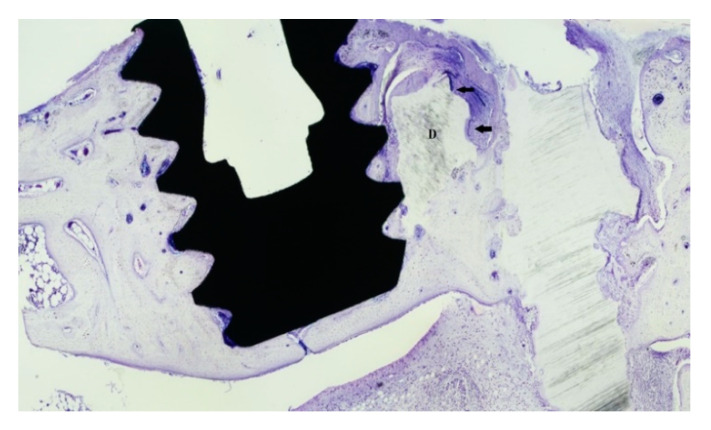
Histological specimen demonstrating a dentin block (**D**) that is fused to the surrounding bone and exhibiting external resorption (arrows). Fragmentary contact between the dentin and titanium surface can be noted. Toluidine blue stain; 10× magnification.

Over time, the dentin grafts were gradually subjected to resorption replacement with bone (Figure 5).

**Figure 6:**
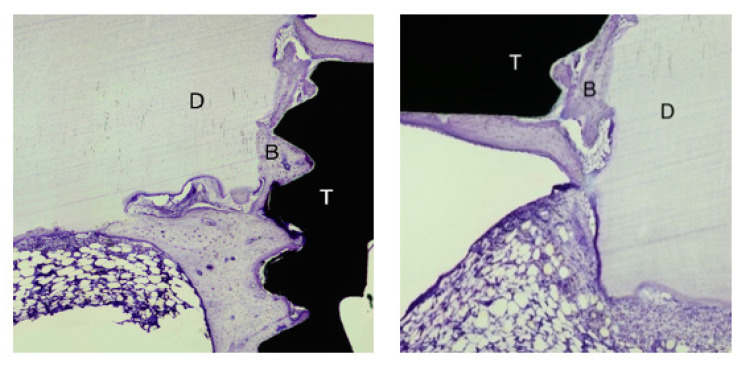
Representative histological sections showing migration of the newly formed bone (**B**) into the spaces between the dentin fragment (**D**) and the titanium surface (**T**). Toluidine blue stain; 4× magnification.

In general, no or scarce direct contact between the xenogenic dentin and the micro-implant surface could be noted after 24 weeks of bone healing. A few osteoclasts could be identified on the surface of the dentin, mostly located adjacent to the present native bone tissue. The dentin particles, regardless of particle size, were otherwise surrounded by fibrous tissue with scarce presence of cells. The dentin material did not seem to induce bone apposition on the implant surface. Instead, newly formed bone seemed to migrate into the micro-gap between the dentin and the titanium surface (Figure 6).

### 3.3. Histomorphometry

A total of 26 specimens were available for histomorphometry analysis. The ROI comprised the first five coronal threads corresponding to the length placed in dentin. The non-demineralized group (*n* = 12) revealed a bone-to-implant contact (BIC) mean of 36.2%. BIC for the mineralized group (*n* = 14) was 40.4% (nonsignificant, *p* = 0.48). The percentages of new bone fill in the area (BA) within the threads (percentage bone fill) for the non-demineralized group were 67.4% and 72.4% (nonsignificant, *p* = 0.09) for the demineralized group. Overall, the BIC and percentage of new bone fill for the demineralized group were higher than the that for the non-demineralized specimens. Only fragmented areas of direct contact between the dentin and the titanium surface could be noted (Figure 5).

## 4. Discussion

The aim of this experimental study was to study the integration of dental implants in conjunction with grafted dentin and to evaluate the morphological appearance and mineral content in decalcified dentin using different demineralization protocols prior to grafting. Overall, limited or no contact between the micro-implants and the xenogenic dentin grafts was demonstrated. The overall BIC and percentage of new bone fill (BA) was in favor of the demineralized group. Furthermore, it was indicated that the dentin granules were encapsulated by fibrous connective tissue in most cases, whereas most of the dentin blocks were fused with bone. Previous studies have shown that if dentin grafts are mobile during the osteogenic phase of bone healing, fibrous tissue is frequently formed, compromising proper graft incorporation [4,24,25]. In contrast, when a dentin-block graft is stabilized within a bone cavity, it is incorporated into the recipient bone at a higher degree [21,26]. This is the most plausible explanation for why dentin granules in the majority of experimental bone cavities are encapsulated by fibrous tissue.

Rabbit tibia were previously shown to be a suitable experimental model compared to jawbone [25]. It was shown that dentin implanted in the tibial cortex were fused directly with bone over large areas, whereas most of the dentin blocks grafted to the mandible were confined to the connective tissue without any bone contact. This is probably due to the thin mandibles and because the soft tissue around the mandible comprises muscles and many mobile structures in a very active area for chewing and swallowing, which may increase the risk of the dentin block being moved after some initial resorption.

Non-demineralized dentin is less prone to bone formation, and its resorption starts later than that of totally demineralized dentin when placed in muscle pockets of rats [14]. On the other hand, completely demineralized dentin may be resorbed too fast, resulting in insufficient bone volumes [14].

Demineralization is a crucial step in dentin grafting [6,27]. This process increases the bioavailability of matrix-associated non-collagenous proteins such as dentin sialophosphoprotein (DSPP), dentin phosphoprotein, osteocalcin, osteonectin, and BMP, which may enhance new bone formation [28,29,30,31]. The purpose of EDTA conditioning was not to completely remove minerals from the dentin but rather to modify the surface facing the surrounding tissue (periosteum and bone surface), as seen in the EDX analysis. Notably, no difference in radiograph contrast was observed after 12 h of conditioning (Figure 2B). The effect of dentin graft surface decalcification on the graft–host response can be speculated upon. Most probably, EDTA conditioning enhances the dentin graft surface exposure of its organic components to the wound area, possibly contributing to rapid initiation of the bone replacement resorption process, where the initial inorganic tissue components are already absent.

EDTA at neutral pH was chosen since it selectively removes minerals from the dentin surface without any adverse effects on the integrity of the collagen matrix. Etchants operating at lower pH have been shown to have a negative impact on the integrity of the collagen matrix, thereby affecting the bone-formation capacity of dentin [32]. It has also been claimed that bone morphogenetic properties are lost if the matrix is exposed to chemical solvents that denature or otherwise derange the three-dimensional framework of the fibrous proteins [19]. EDTA, however, seems to preserve the integrity of intercellular structures [33].

The BIC and BA values were slightly higher for the demineralized group. However, this difference was proven not be statistically significant, implying that EDTA conditioning of the dentin grafts for 12 h did not further improve the bone-inductive capacity of the dentin grafts. One might speculate about the duration, conditioning agent, or even the need for conditioning and whether a longer period of demineralization, alternative conditioning agent, or conditioning in general would result in a different healing pattern.

After preparation, the dentin grafts were placed in chlorhexidine for 60 min to reduce bacterial growth and were thereafter stored dry for one month before implantation. In vitro studies have proven that chlorhexidine is toxic to fibroblasts and odontoblast-like cells [34,35]. One might speculate about the possible effects on bone healing due to chlorhexidine pretreatment. However, the same processing protocol has been used in previous studies without any adverse effects on healing of the dentin grafts [36,37,38]. Radiation, heat, and ultrasonic vibration may reduce the bone inducing capacity of dentin. Therefore, the dentin implants in this study were sterilized only by placing them in chlorhexidine.

The soft and hard tissue healing in the rabbits participating in the study was uneventful, without any macroscopic or microscopic signs of infection or inflammation. The immunogenic properties of a tooth are mostly related to the periodontal ligament and pulp, which had been removed in this study [37]. However, the presence of non-collagenous proteins within the dentin grafts can play a role in activation of the immune response at the beginning of the bone replacement resorption process.

It was shown in a previous work comparing mineralized with demineralized dentin that a larger number of sites in a demineralized group exhibited inflammation. Hence, it was speculated that a dentin-demineralization procedure might expose more of the matrix, inducing a higher rate of inflammation. Remnants of EDTA could be the reason for this phenomenon, indicating a need for a more elaborate cleaning procedure [21]. In this study, no inflammatory cells in any ROIs in any sample were observed.

The dentin grafts exhibited all the characteristics of human dentin. In both longitudinal and transversal sections of the dentin grafts, the tubules were clearly discernible (Figure 4A,B). In a close up of transversally sectioned dentin, the effect of the surface EDTA conditioning is evident, showing a rough surface appearance of the inter-tubular dentin along with smooth edges around the tubule openings.

In the elemental analyses of EDTA-treated dentin, carbon (C) and nitrogen (N) were used as markers for the organic components. The organic part was primarily based on carbon-based molecules. Carbon was the most abundant element in the organic part of dentin. Nitrogen is considered a marker for proteins, more specifically the amino parts of peptide bonds. Nitrogen was the second-most abundant element in dentin, indicating high levels of proteins on EDTA-treated dentin surfaces.

The levels of the hydroxyapatite constituent’s calcium (Ca), phosphorus (P), and oxygen (O) were low on the EDTA-treated dentin graft surfaces, with phosphorus being completely absent. A minor part of the inorganic part of dentin is in fact carbonated hydroxyapatite, but to a very low extent. The carbon revealed in the EDX measurements of the dentin grafts are considered of organic origin.

## 5. Conclusions

The conclusions of this study are as follows:

No implants were integrated in direct contact with the dentin particles or blocks. EDTA conditioning did not completely decalcify the dentin grafts. The dentin graft surfaces were almost devoid of calcium and were completely devoid of phosphorus. Rigid fixation of the dentin grafts was a prerequisite for proper bone healing. Non-fixated dentin granules were encapsulated by connective tissue. EDTA conditioning of the dentin grafts did not further improve the bone inductive capacity as indicated by BIC and BA.

The impact of dentin as a bone substitute material mostly relies on a replacement resorption pattern. Further studies are needed in order to fully elucidate the importance of dentin graft pretreatment or conditioning.

## Figures and Tables

**Table 1:** Energy dispersive X-ray analysis (EDX) of the decalcified dentin surfaces. Within the window of analysis, four random locations were measured. For each location, an elemental analysis spectrum was obtained. The means and SD for each spectrum and element are presented.

	C	N	O	P	Ca	Au
**Spectrum 1**	57.95	23.68	14.70	−0.35	0.16	3.85
**Spectrum 2**	52.97	28.23	15.09	0.10	0.48	3.13
**Spectrum 3**	55.11	27.28	15.30	0.04	0.18	2.08
**Spectrum 4**	54.40	27.18	14.47	0.21	0.29	3.35
**Mean**	55.13	26.59	14.89	0.00	0.28	3.10
**Std.deviation**	2.08	2.00	0.38	0.24	0.14	0.75
**Max**	57.95	28.23	15.30	0.21	0.48	3.85
**Min**	52.97	23.68	14.47	−0.35	0.16	2.08
